# A novel quantitative and reference-free ultrasound analysis to discriminate different concentrations of bone mineral content

**DOI:** 10.1038/s41598-020-79365-0

**Published:** 2021-01-11

**Authors:** A. Sorriento, A. Poliziani, A. Cafarelli, G. Valenza, L. Ricotti

**Affiliations:** 1 The BioRobotics Institute, Scuola Superiore Sant’Anna, 56127 Pisa, Italy; 2 Department of Excellence in Robotics & AI, Scuola Superiore Sant’Anna, 56127 Pisa, Italy; 3 Bioengineerring and Robotics Research Centre E Piaggio, University of Pisa, 56122 Pisa, Italy; 4 Department of Information Engineering, University of Pisa, 56123 Pisa, Italy

**Keywords:** Diseases, Engineering

## Abstract

Bone fracture is a continuous process, during which bone mineral matrix evolves leading to an increase in hydroxyapatite and calcium carbonate content. Currently, no gold standard methods are available for a quantitative assessment of bone fracture healing. Moreover, the available tools do not provide information on bone composition. Whereby, there is a need for objective and non-invasive methods to monitor the evolution of bone mineral content. In general, ultrasound can guarantee a quantitative characterization of tissues. However, previous studies required measurements on reference samples. In this paper we propose a novel and reference-free parameter, based on the entropy of the phase signal calculated from the backscattered data in combination with amplitude information, to also consider absorption and scattering phenomena. The proposed metric was effective in discriminating different hydroxyapatite (from 10 to 50% w/v) and calcium carbonate (from 2 to 6% w/v) concentrations in bone-mimicking phantoms without the need for reference measurements, paving the way to their translational use for the diagnosis of tissue healing. To the best of our knowledge this is the first time that the phase entropy of the backscattered ultrasound signals is exploited for monitoring changes in the mineral content of bone-like materials.

## Introduction

Ultrasound (US)-based techniques are an attractive option in the field of tissue diagnosis since they are non-invasive, safe, portable and low cost. Conventional B-mode images are widely used as a clinical diagnostic tool in radiology. However, they do not guarantee a quantitative, system-independent metrics to characterize tissues. Indeed, grayscale signal-based analyses are both system- and operator-dependent, being affected by a variety of factors that are not all associated with specific tissue properties. Moreover, B-mode US images contain less information than the raw radio-frequency (RF) data of US backscattering signals, due to many processing steps involved in the analog-to-digital conversion and image formation.

Quantitative ultrasound (QUS) techniques offer the advantage of working directly on RF data extracting objective and quantitative metrics for tissue characterization^1^. QUS imaging techniques include spectral-based parameterization^2^, elastography^3^, shear wave imaging^4^ and envelope statistics^5^. In recent years, spectral-based parameterization and envelope statistics showed successful outcomes in many applications, even if they are not available in conventional US machines, typically used in the clinics^1^.

Spectral parametrization grounds on the analysis of the normalized power spectrum of RF signals for deriving information about tissue microstructure. The normalized power spectrum is calculated by dividing the tissue power spectrum by a reference spectrum using a planar reference method in case of single-element transducers or a reference phantom technique for clinical array systems^1^. Hence, a reference signal is always needed during the acquisitions in order to remove artifacts and dependency from the system. The extracted parameters typically include the backscatter coefficient, the slope and intercept of the regression line as well as the mid-band fit (MBF). The spectral slope has been shown to correlate with the scatter size, while the MBF depends on the size, concentration and relative acoustic impedance of the scattering elements^6^. Spectral-based parametrization has been widely used in the field of tissue engineering for the estimation of cell concentration^7^ and osteoblast differentiation^8^.

On the other hand, the envelope statistics grounds on the concept that the shape and attributes of the backscattered US envelope also contain information about tissue microstructural properties. Several statistical models for analyzing the envelope have been proposed, and one of the most used is the Nakagami distribution^5^. Shannon entropy has also been applied to describe changes in scattering media. To find differences in entropy distribution, US entropy imaging techniques have been employed. Typically, the entropy is estimated on the part of the envelope images that are derived from the absolute value of the Hilbert transform of backscattered RF signals.

QUS techniques have been exploited, with a certain success degree, to improve medical diagnostics of soft tissues, such as classification of tumors and lymph nodes, detection of cancer and liver diseases, monitoring therapies^1^ and cell death^6^. However, very few works tried to extend QUS techniques to orthopedic applications and, in particular, for the assessment of bone fracture healing, despite there is evidence that the US can detect callus formation before radiographic changes become visible^9^.

Fracture healing is a complex and dynamic process, which involves both biological and mechanical aspects^10^. Although the assessment of bone union is of key importance, there are no standardized methods in the clinical orthopedics to do that^11–13^. Currently, radiographic and clinical examination are the most used methods for monitoring bone healing. Imaging tools such as computed tomography, ultrasonography, positron emission tomography and magnetic resonance imaging can also be employed for the characterization of the geometry and the microstructure of the bone, even if they do not offer information on the state of strength and composition^14^. Moreover, all the available methods are subjective, operator-dependent and non-quantitative. A reliable tool able to establish an objective endpoint of healing would be desirable for a precise and patient-specific diagnosis. Such an indicator could improve the outcome of healing, determining: (1) early detection of impaired healing, (2) the need for further treatment or operation to promote healing and (3) the formulation of patient-specific rehabilitation protocols.

QUS techniques have been investigated to assess bone mineral density in patients affected by osteoporosis diseases^15,16^ and rheumatoid arthritis^17^. On the other hand, a few studies have been conducted in the context of bone fracture, with most of them only focused on evaluating the speed of sound. The propagation velocity across fractured bones has been explored as an indicator of healing in animal and clinical trials^18^. However, due to a lack of standardization in the procedures, it is challenging to provide reference velocity and attenuation values for each stage of healing. Al-Nashash et al.^19^ proposed the image intensity as a metrics for monitoring the healing of fractured limbs by assessing changes in acoustic impedance. Results of a pilot study on four patients showed the potential of image intensity to quantify the stage of bone healing. However, further assessments and applications of this methodology are not available in the literature. Moreover, a few groups explored the quantitative characterization of constructs in the field of orthopedic tissue engineering. Gudur et al*.*^20^ validated high-resolution spectral parameterization as a method to characterize a developing mineral phase in three-dimensional collagen hydrogels. Spectral parameters extracted from a calibrated power spectrum of RF signals were able to identify hydroxyapatite concentrations, particle size and mineral distribution. Mercado et al*.*^21^ showed the capability of the integrated backscatter coefficient (IBC) to recognize different densities and diameters of collagen fibers in three-dimensional collagen gels. However, in all the mentioned studies, a reference signal is always needed to normalize the tissue power spectrum and remove artifacts from the systems. In view of an in-vivo translation of this measurement, the clinical ultrasound device has to be equipped with a reference phantom with known acoustic properties in order to calibrate the system, thus performing a time-consuming and vulnerable to errors procedure before extracting the spectral parameters. Moreover, this method has specific requirements for the acoustic properties of the reference phantom: its speed of sound and attenuation must be similar to those of the investigated tissue^22^.

To the best of our knowledge, nobody has systematically studied the acoustic contributions of the single mineral components of the bone during the healing phases. We hypothesized that both phase and amplitude information of RF ultrasound signals could provide objective metrics for discriminating different concentrations of hydroxyapatite (HA) and calcium carbonate (CaCO_3_), which are the main components of the bone mineral extracellular matrix. Notably, in this study, we proposed a novel index extracted from raw RF data, without the need for a normalization step with respect to a reference signal, able to discriminate changes in HA and CaCO_3_ concentrations in bone-mimicking phantoms.

## Materials and methods

### Phantom preparation

Agarose-based hydrogels were chosen as an ultrasonically-neutral matrix in which adding the inorganic components of interest. They were prepared by dissolving low-melt agarose powder (9414, Sigma Aldrich) in deionized water at a concentration of 2% w/v. Solutions were kept at 60 °C for 1 h under continuous stirring and then cooled down at room temperature to allow material reticulation. HA and CaCO_3_ were then included in the agarose matrix at different concentrations to reproduce the inorganic phase of bone callus during the healing process. The maximum concentrations of HA (50%) and CaCO_3_ (6%) reflected the ones found in the healthy human bone^23,24^.

Composite agarose-HA hydrogels were fabricated, adding HA powder (900204, Sigma Aldrich) to the agarose solution, yielding a final concentration of 10%, 20%, or 50% w/v. The solution made of agarose and HA powder was kept in an ultrasonic bath for 30 min at 60 °C to promote a homogenous particle dispersion. Then, the mixed solution was cooled at room temperature and stored at 4 °C.

Composite agarose-CaCO_3_ hydrogels were prepared starting from a surface coating of CaCO_3_ particles, by using glycol-chitosan (GC) (G7753, Sigma Aldrich). This step was needed to improve particle dispersion in the aqueous solution^25^. Solutions of GC at different concentrations (0.1%, 0.5% and 1% w/v) were prepared by adding GC powder to deionized water under continuous stirring for 1 h. First, to evaluate the dispersion ability of such different concentrations, CaCO_3_ powder (C6763, Sigma Aldrich) was added to the GC solution at a concentration of 4% w/v. The compound was sonicated through an ultrasonic homogenizer Sonopuls HD 4050 (Bandelin, Berlin) for 30 min in pulse mode (30 s on and 30 s off) at 35% of the maximum power. Then, visual inspection allowed assessing the presence or absence of particle precipitation, 30 min after sonication. The deposition of particles on the bottom surface of the vial was not observed even for the highest concentration of GC (1% w/v), as reported in Figure S1 of the supplementary material.

To prepare the target composite agarose-CaCO_3_ hydrogels, the CaCO_3_ powder was added to the GC solution achieving particle concentrations of 4%, 8%, or 12% w/v. Then the compound was sonicated as described before. Finally, the GC solution with CaCO_3_ particles was mixed with the agarose solution, yielding composite agarose-CaCO_3_ samples at concentrations of 2%, 4%, or 6% w/v.

**Figure 1:**
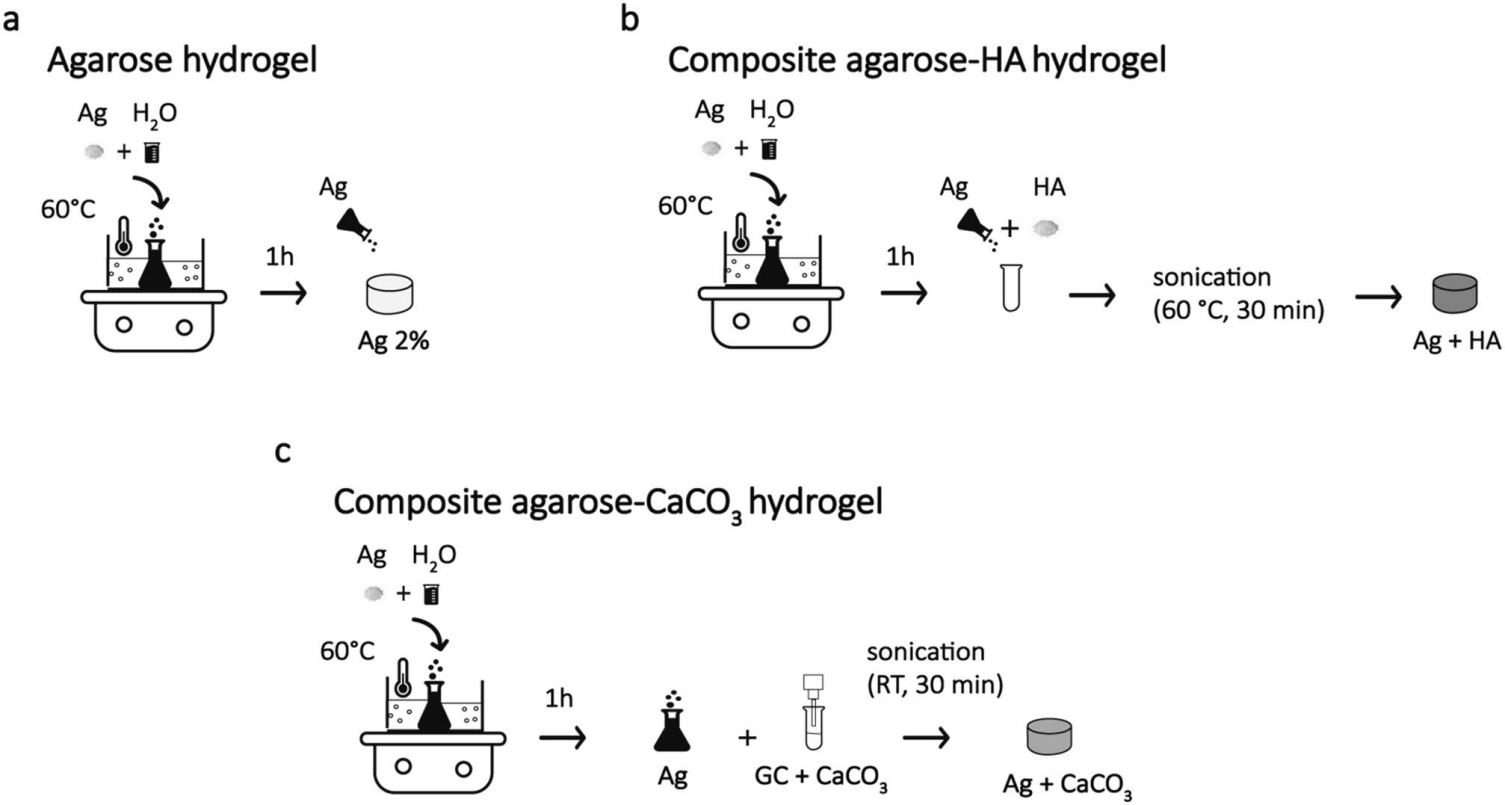
Phantom preparation process. The procedure used for phantom preparation is depicted for agarose hydrogels (**a**), agarose-HA hydrogels (**b**) and agarose-CaCO_3_ hydrogels (**c**). Agarose (Ag), Hydroxyapatite (HA), Calcium carbonate (CaCO_*3*_).

A schematic representation of the sample preparation procedure is depicted in Fig. 1.

### Experimental setup for US data acquisition

**Figure 2:**
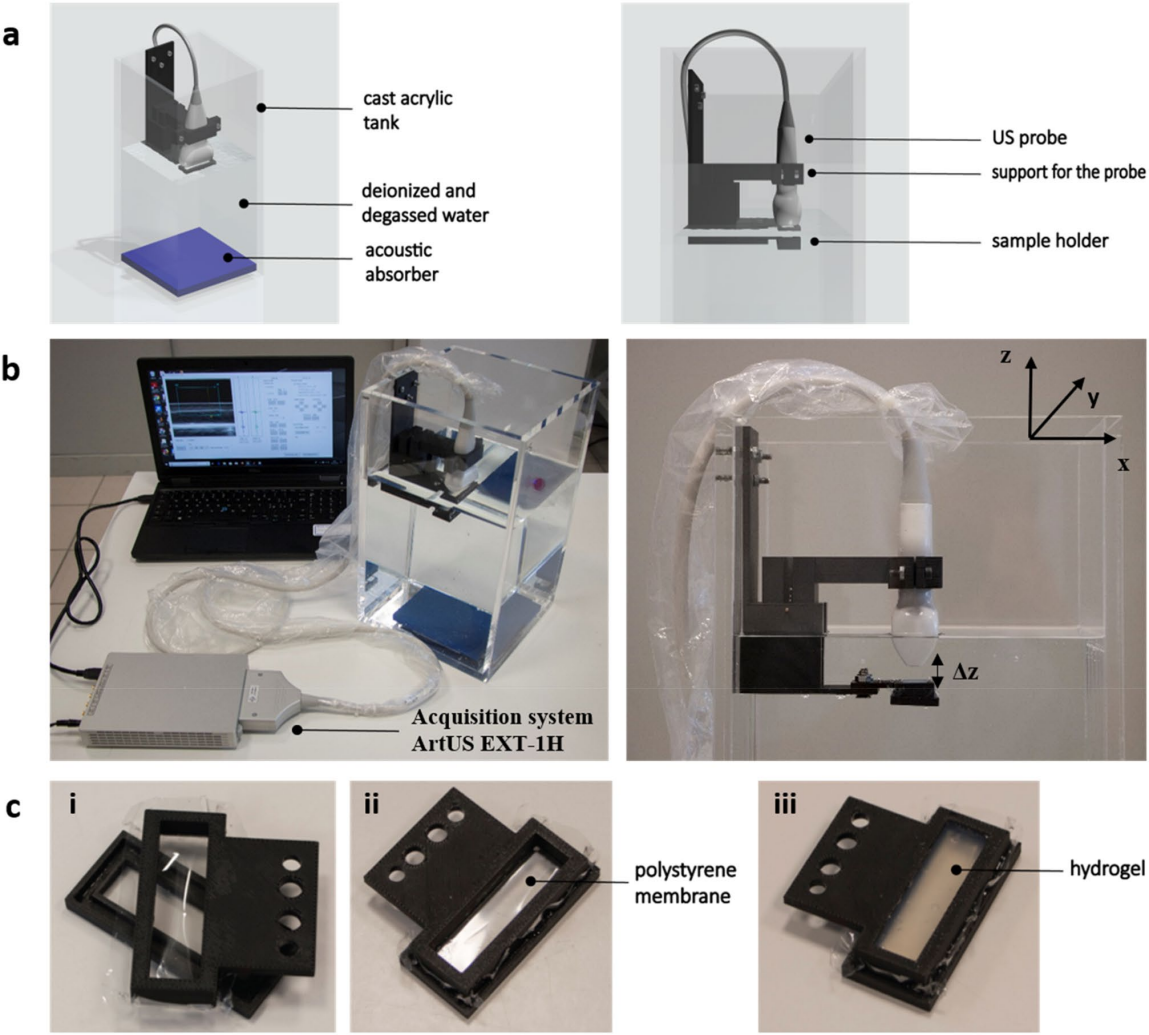
Experimental setup for US acquisition. (**a**) Schematic representation of the setup; (**b**) images of the real experimental setup, including the acquisition system and the pc; (**c**) structure of the sample holder: in i) the two interlocking parts and the polystyrene membrane are shown, in ii) the assembled system is reported and in iii) the sample holder also containing a hydrogel is shown.

The experimental setup used to acquire US data from the different hydrogels is shown in Fig. 2.

Figure 2a shows a schematic representation of the experimental setup, which includes a tank filled with deionized and degassed water at room temperature, a support for the US probe and a sample holder. The support for the probe and the sample holder were printed using a M200 Plus 3D printer (Zortrax, Poland), choosing acrylonitrile butadiene styrene (ABS) as the material. Then, the single components were mounted and fixed to the tank through nuts and screws. A high-frequency acoustic absorber (Aptflex F28, Precision Acoustics, UK) was used to prevent signal reflection from the bottom of the tank, which may affect the acquisition. As shown in Fig. 2c (panel i), the sample holder was composed of two interlocking parts with a polystyrene film (thickness: 29 µm) in the middle (panel ii) to hold up the sample poured on such a membrane (panel iii). The hydrogel-containing holder was fixed through screws to the support in the water tank Fig. 2b, for US measurements.

US measurements were carried out using an ArtUS EXT-1H system (Telemed, Italy) equipped with a 192 elements linear probe L15-7H40-A5 working in the frequency range 7.5–15 MHz. The acquisition of raw RF data was performed using a dedicated software interface developed by the company. The interface allowed setting the US scanner parameters, such as scanner power, scanning depth, focus, transmission frequency, size and position of the acquisition window and collecting RF data for off-line analyses. First, the probe was moved in the z-direction (see Fig. 2B) to set the distance (Δz) between the sample and the probe, allowing to get the best resolution in B-mode images. A distance of 1 cm was found as the most suitable one for all the samples and was thus fixed for subsequent analyses. Data were collected at 35% of the scanner power, with a scanning depth of 20 mm and a transmission frequency of 15 MHz, adjusting the focus in the middle of the samples. The scanning parameters and the acquisition window were fixed in the software interface and used over all the experimental sessions. Backscattered RF measurements from each hydrogel in the sample holder were conducted. Five independent samples were analyzed for each hydrogel type. For each sample, five measurements were carried out, by removing and then placing the holder and the sample in the setup again before each measurement, to take into account possible variations in sample holder positioning. After RF acquisition of Ag 2% samples, a reference RF acquisition of the polystyrene film without any sample was also made, to validate the setup.

### Validation of the experimental setup

**Figure 3:**
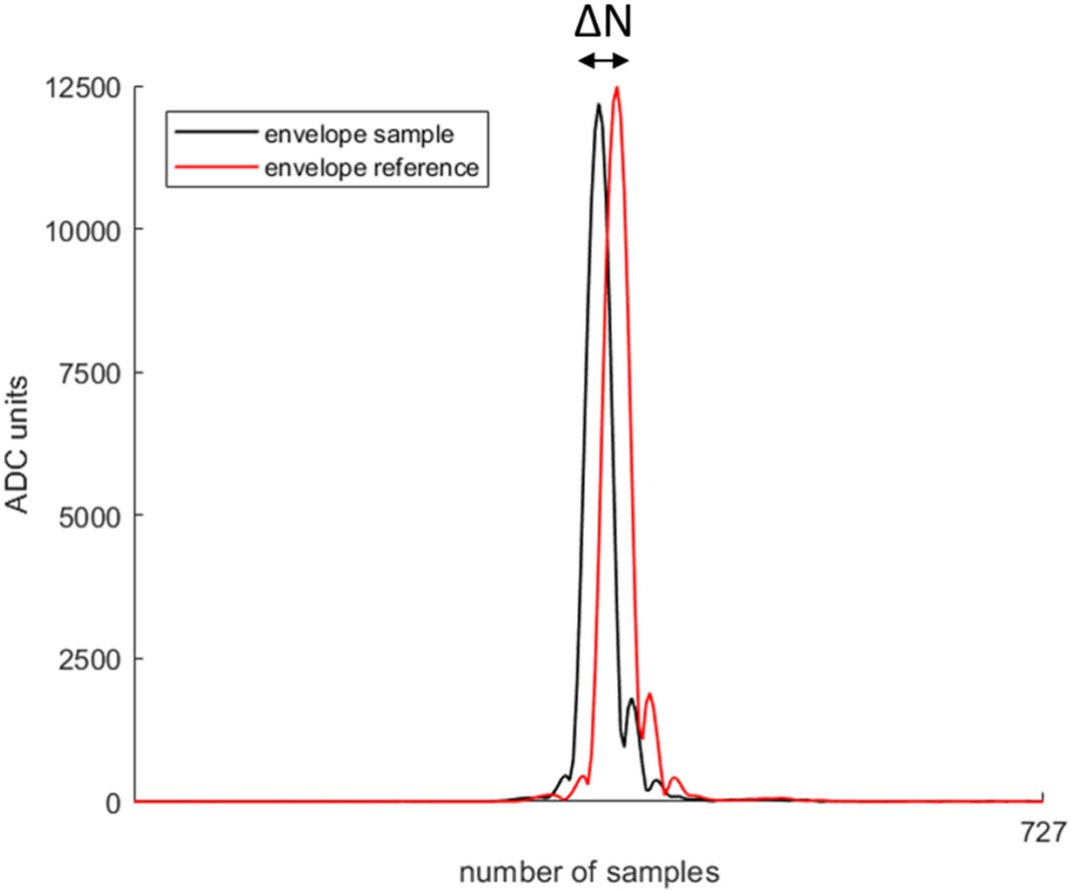
Example of signal envelopes for SoS measurement of Ag 2% samples. The envelopes of the signal in the presence of the sample (black line) and in the absence of the sample (red line) are shown in the figure for the central scanning line.

Before starting the experimental session, a validation of the experimental setup was performed by comparing the speed of sound (SoS) calculated on a known material (Ag 2% samples) and the ones available in the literature^26^. Three independent agarose samples were tested and five different measurements for each sample were performed. Agarose SoS was calculated through Eq. (1)

1 $${SoS}_{s}=\frac{2*d}{\frac{2*d}{{SoS}_{w}(^\circ T)}-\Delta t}$$

where *SoS*_*s*_ is the speed of sound of the sample, *d* is the sample thickness, *Δt* is the delay between the time of flight measured in the presence of the sample and the time of flight measured in the absence of the sample, and *SoS*_*w*_ is the speed of sound of water, which depends on temperature, as reported by Marczak et al*.*^27^*.* For each sample, the thickness was measured through a caliber. The cross-correlation between the envelope signal from the sample and the envelope signal from the reference (i.e., the polystyrene membrane) was calculated for each scanning line. The lag in terms of the number of samples corresponding to the maximum cross-correlation signals was considered as ΔN. The time shift *Δt* between sample and reference signal was obtained by dividing ΔN for the sampling frequency of the acquisition system (i.e., 40 MHz). For each sample and each repetition, the average value of SoS_s_ was calculated along the 101 RF lines. To exclude the outliers, we used the median absolute deviations (MAD) technique, according to which the values more than three scaled MAD from the median were removed. Finally, a single value of SoS_s_ was calculated by averaging fifteen values, obtained from the three samples and the five measurements done on each sample. An example of the signal envelopes used for SoS measurement acquired in the presence and the absence of the sample for the central scanning line is shown in Fig. 3.

The results showed a SoS value of 1494 ± 6.8 m/s, which is in line with previous results reported in the literature (1490 ± 2.0 m/s) obtained through a different experimental setting^26^. This demonstrated the ability of the proposed set up to investigate the acoustic properties of known materials in a reliable and repetitive way, due to a smart design able to reduce artifacts and reflection not associated with the sample.

### SEM imaging and EDX spectroscopy

Scanning electron microscopy (SEM) and energy dispersive X-ray (EDX) spectroscopy were used to analyze the morphology and content of each sample, respectively. Immediately after performing US measurements, samples were fixed and dehydrated, as reported in^28^. Samples were immersed in a 4% w/v paraformaldehyde solution (30 min) and then in a 2.5% w/v glutaraldehyde solution (30 min) at room temperature. Then, samples were dehydrated through an ethanol gradient (0%, 25%, 50%, 75% and 100%, 10 min for each ethanol concentration), dried overnight and gold-sputtered. Both SEM and EDX analyses were conducted using a dual-beam microscope Helios (Hillsboro, OR, USA). SEM scans were carried out by setting a beam voltage of 5 kV and a current of 43 pA, while for EDX analysis, a beam voltage of 15 kV, a current of 0.17 nA, and an acquisition time of 90 s were set. SEM images were taken at two magnifications (700X and 2400X), while EDX analysis was performed at 700X.

### RF data processing

As mentioned, five independent samples were analyzed for each concentration and three concentrations for each group of mineral components were considered. Overall, fifteen samples of composite agarose-HA hydrogels and fifteen samples of composite agarose-CaCO_3_ hydrogels were tested. Five independent samples of agarose hydrogels were also included as a control. For each sample, five measurements were performed, as previously described (see “Experimental setup for US data acquisition”).

RF data were processed off-line in the Matlab environment. Since the measurements were made in static conditions and we were not interested in motion-related effects, a single RF frame was acquired for all the analyses. Each recorded RF frame resulted in a matrix in which the columns (101) represented the number of RF scanning lines in a specific RF window, while the rows (727) constituted the number of samples in a single scanning line, with a sampling rate of 40 MHz. B-scan images were then generated by calculating the grayscale envelope and log-compression of the RF signal.

To take into account the quantity of energy reflected at the first interface (between water and sample) and at the second interface (at the polystyrene membrane), the maximum amplitude value of the RF signal at the two interfaces was calculated for each RF line of each data matrix. Then, a mean amplitude value along all the RF lines was computed for the first and the second interface, obtaining the indices peak1 and peak2, respectively.

The instantaneous phase signal was calculated from the imaginary part of the Hilbert transform for each RF line of each sample. The phase of RF backscattered signal includes information on the characterization of the scattering medium. In particular, it is known that scatters can be detected by monitoring variations in the phase profile of the RF signal^29^. Hence, we aimed at investigating the degree of irregularity of the instantaneous phase series by computing the sample entropy (SampEn)^30^ of the phase signal in bone mimicking materials. Indeed SampEn is a measure of the predictability of a series and it is defined as the negative natural logarithm of the conditional probability that two sequences that are similar for m points, also match at the subsequent point, according to Eq. (2):

2 $$SampEn\left(m,r,N\right)=-\mathrm{log}\frac{A}{B}$$

where *m* (named embedding dimension) is the length of the sequences to be compared, *r* is the tolerance for accepting matches, *N* is the length of the time series, whereas *B* and *A* are the probabilities that two sequences in the input are similar for *m* and *m* + 1 points respectively. In order to investigate irregularities in time-series, a single SampEn parameter was calculated for each RF line, setting the embedding dimension (*m*) to 3 and the tolerance (*r*) to 0.2 times the standard deviation of the original signal. Then, a mean along the RF lines was computed in order to manage one index for each measurement made on each sample.

A combination of the parameters peak1, peak2 and SampEn was investigated according to Eq. (3):

3 $$f\left(s\right)={(SampEn)}^{4}\times \frac{peak2}{peak1}$$

where *s* is the backscattered signal, SampEn is the sample entropy of the phase signal calculates as in (2), and peak1 and peak2 are the average amplitude values at the first and second interface, respectively.

This model aims at concurrently quantifying the degree of predictability of the instantaneous phase signal (through the SampEn parameter) together with the US energy that crosses the sample (through the term $\frac{peak2}{peak1}$).

### Statistical analyses

Group-wise descriptive statistics were expressed as median ± interquartile range and graphically shown as boxplots. Non-parametric statistical differences were investigated between seven samples including CaCO_3_ 2%, CaCO_3_ 4%, CaCO_3_ 6%, HA 10%, HA 20%, HA 50%, and Ag 2% (control). Each sample comprises 25 estimates from the five measurements performed on the five independent material samples. Because of the non-normality of data distribution (SampEn values are always positive; therefore they are Non-Normally distributed), the statistical comparison between all samples was performed using a non-parametric Kruskal–Wallis test, whereas a post-hoc test between samples was performed using non-parametric Mann–Whitney test for unpaired data. Statistical significance was corrected for multiple comparisons following the Bonferroni–Holm rule, and a corrected p value lower than 0.05 was deemed as significant.

## Results

### SEM imaging and EDX spectroscopy

**Figure 4:**
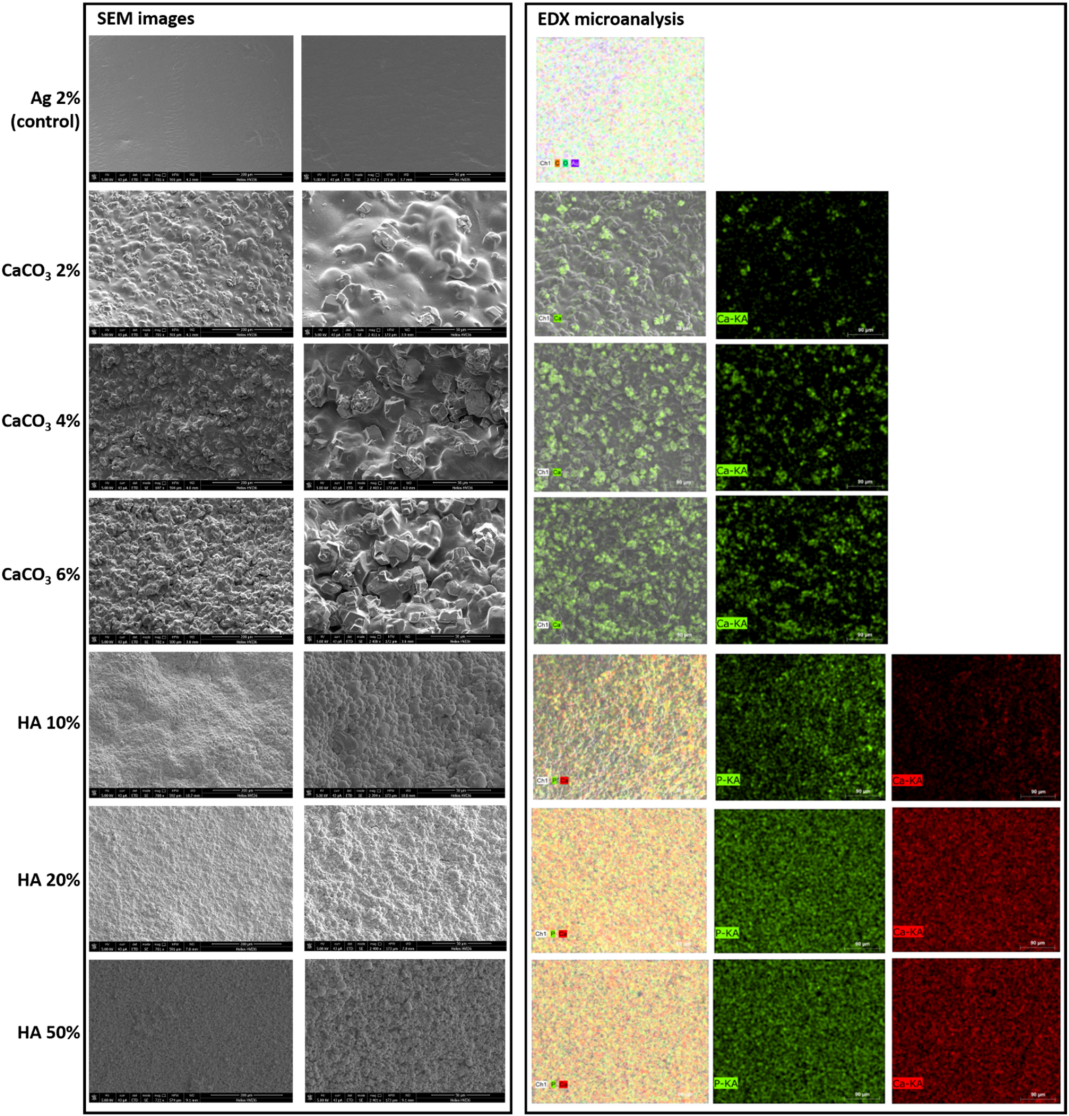
SEM images and EDX analysis for samples containing different concentrations of CaCO_3_ and HA particles. SEM images (left panel) are reported at 700X (first column) and 2400X (second column) magnifications. EDX microanalysis (right panel) is reported at 700X magnification for agarose matrix (2% w/v) and for hydrogels doped with CaCO_3_ and HA particles at different concentrations. For EDX microanalysis, the first image (Ag 2% w/v) reports the main elements automatically found by the system, merged with the SEM image channel; the other images show the single elements analyzed. For samples doped with CaCO_3_ particles, Ca is reported in green. For samples doped with HA particles, P is reported in green and Ca is reported in red. In the agarose matrix without particles, no traces of Ca and P were found, but only C, O and Au.

The morphology and content of one representative sample for each sample type, obtained through SEM and EDX analyses, are shown in Fig. 4.

SEM images at two magnifications (700X and 2400X) are reported in Fig. 4 (left panel), showing peculiar microstructural properties for each sample. The images demonstrated a homogeneous distribution of particles in the agarose matrix, thus demonstrating the suitability of the techniques used for both HA and CaCO_3_ particle dispersion. Agarose matrices with higher concentrations of particles appear more compact and regular, whereas samples at low concentrations present a higher discontinuity in their microstructure.

The EDX microanalysis (Fig. 4, right panel) confirmed the expected changes in particle contents, correlated with the dopant concentrations. Images of composite agarose-CaCO_3_ hydrogels showed a homogenous distribution of particles inside the agarose matrix and an increased content of calcium (Ca) as a consequence of the higher particle concentration. In composite agarose-HA hydrogels, the changes in Ca and phosphorus (P) content were analyzed and were also coherent with increasing particle concentrations. The agarose matrix, without any type of particles, did not present traces of Ca and P, as expected.

### B-mode images

In Figure S2 (Supplementary Material), B-mode images of one representative sample at different transmission frequencies were shown. A slight change in the resolution was observed with increasing the transmission frequency and a more focused image was obtained using data acquired at 15 MHz. As a higher frequency is associated with a higher resolution, we decided to choose RF data acquired with the transmission frequency of 15 MHz for all the analyses.

**Figure 5:**
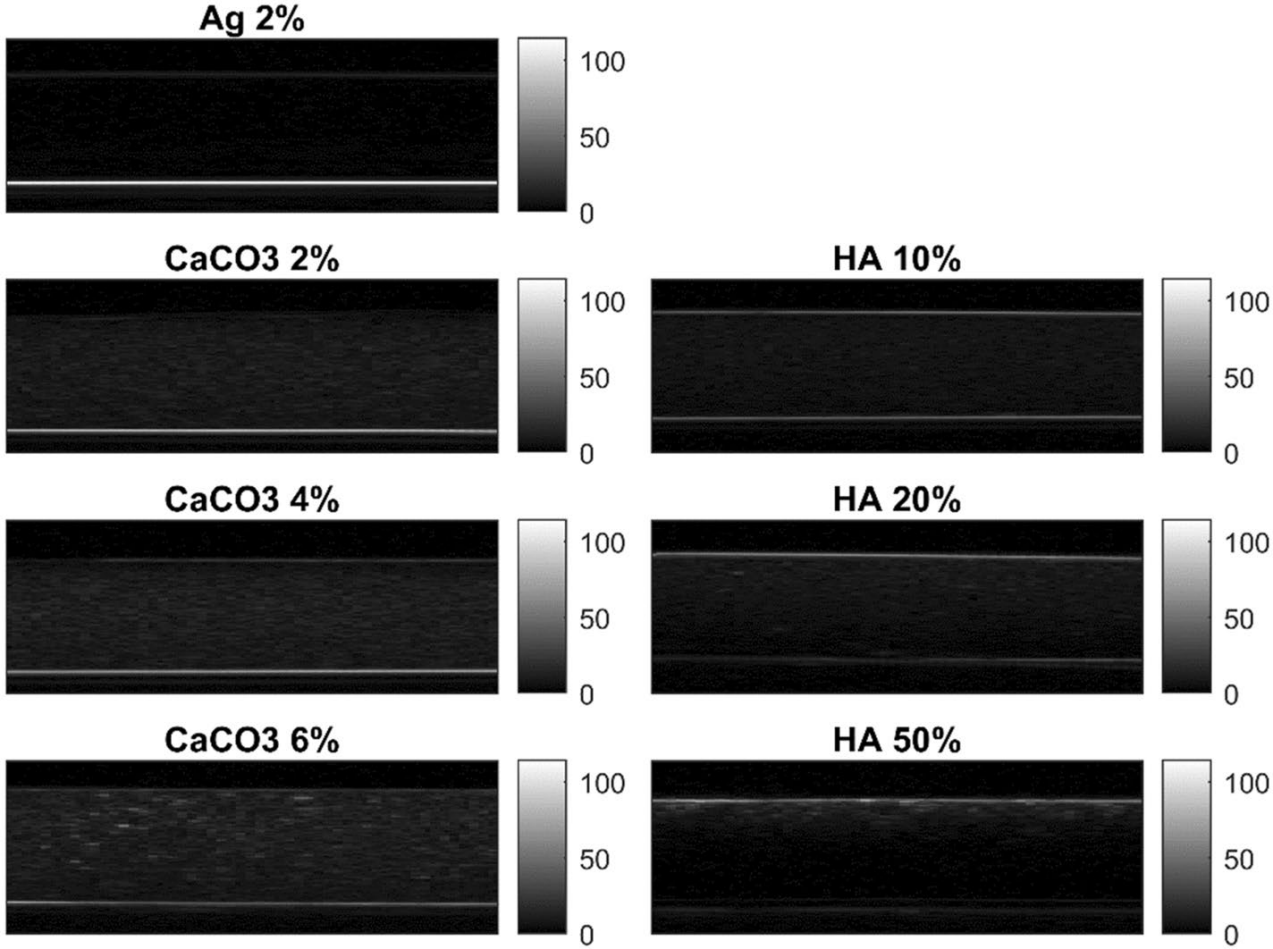
B-mode images of hydrogels. Representative B-scan images of agarose gels without particles (Ag 2%), and with particles at different concentrations: CaCO_3_ 2%, CaCO_3_ 4%, CaCO_3_ 6%, HA 10%, HA 20% and HA 50%. Data were acquired using a transmission wave frequency of 15 MHz. For each image, the color bar shows the pixel brightness.

Representative B-scan images of composite agarose hydrogels are shown in Fig. 5. It can be observed that two bright horizontal lines were displayed in each B-scan image. The top horizontal line corresponded to the US wave reflection at the water—sample top interface due to the mismatch in acoustic impedance of these materials. The bottom horizontal line corresponded to the reflection due to the interface at the polystyrene membrane. The echogenicity of the first horizontal line increased with increasing particle concentration, thus indicating a higher reflection index. Coherently, the echogenicity of the second horizontal line decreased with increasing particle concentration due to a higher energy attenuation within the material. B-scan images provided only a qualitative visualization of particle distribution within the agarose gels.

### RF data analysis

**Figure 6:**
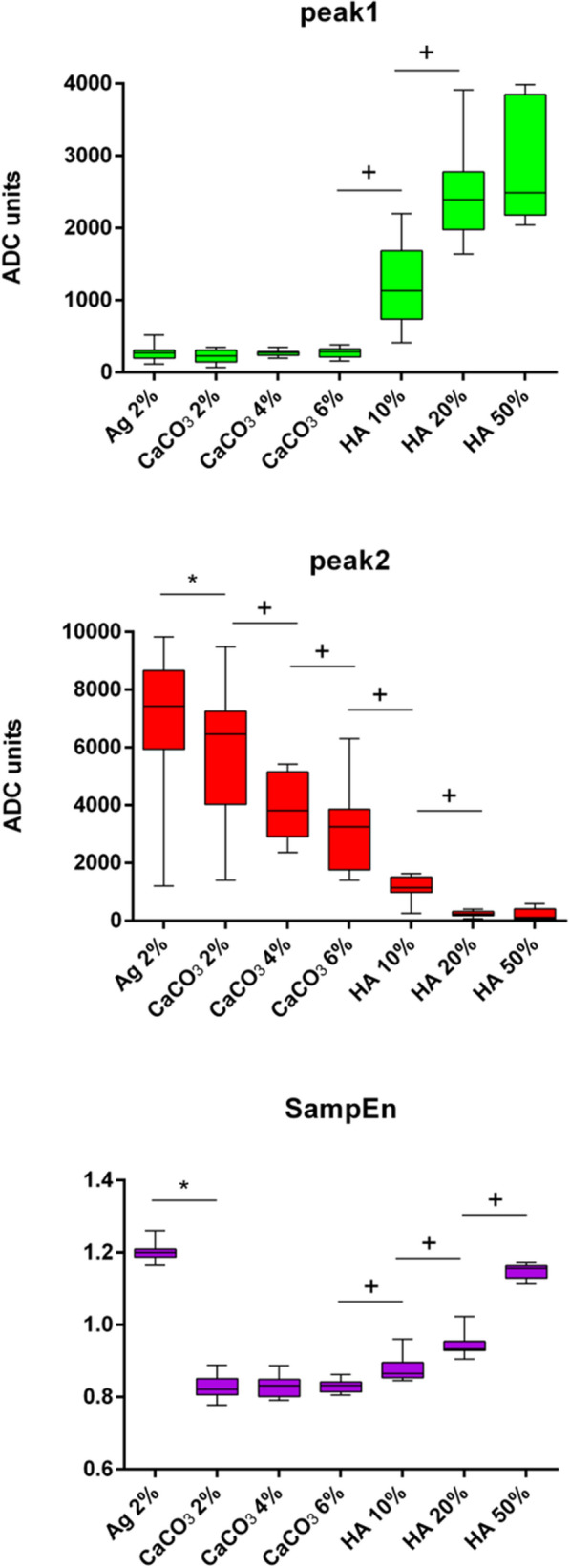
Boxplot statistics for the reflection at the first interface (peak1), the reflection at the second interface (peak2) and SampEn for samples related to different concentrations of CaCO_3_ and HA particles. The symbol * indicates p < 0.05 for the comparison of the groups to the control group, the symbol + indicates p < 0.5 for the comparison of the groups to each other.

Figure 6 displays the boxplot statistics for the three parameters extracted from the RF data analysis: the sample entropy (SampEn), the reflection peak at the first interface (peak1) and the reflection peak at the second interface (peak2). peak1 showed an increasing trend concerning the concentration of particles, thus showing a higher reflection in the case of higher concentrations. Conversely, peak2 decreased when the particle concentration increased. Indeed, as the concentration increases, less energy crosses the sample and thus, less energy is reflected back from the second interface. Thirdly, the SampEn parameter showed an increasing trend with respect to the content of particles, except for the control value (Ag 2%, which has no embedded particles). The SampEn value for the control group was associated with the highest SampEn value, thus indicating the highest irregularity in the instantaneous phase signal of RF data from agarose samples not doped with particles.

**Figure 7:**
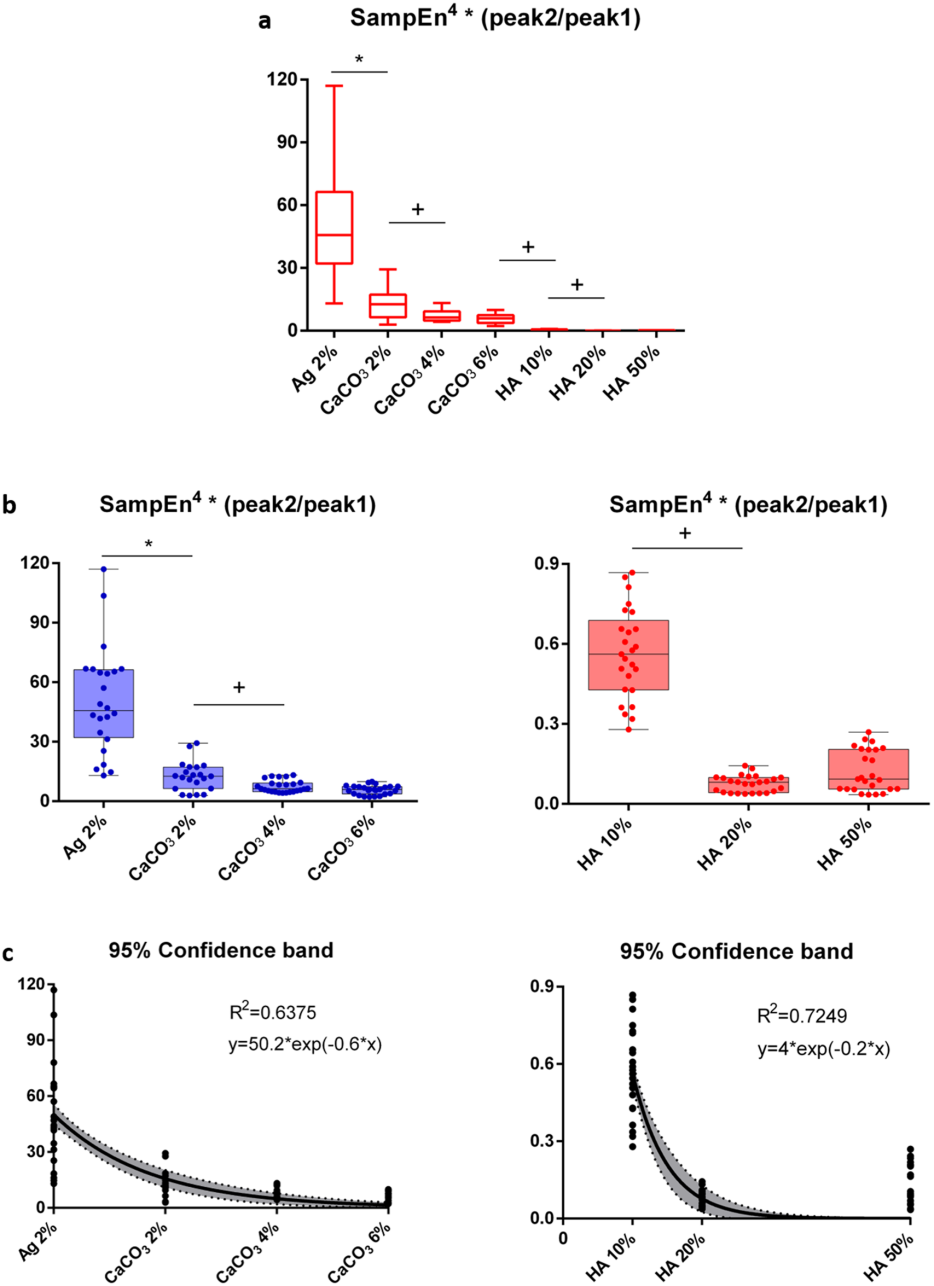
Boxplot and regression analysis of f(s) for samples containing different concentrations of CaCO_3_ and HA particles. The panel (**a**) shows the trend associated to the model including all the previous parameters (SampEn, peak1 and peak2) for: Ag 2% (control), CaCO_3_ at different concentrations (2%,4% and 6% w/v) and HA at different concentrations (10%, 20% and 50%). The statistical comparisons of the closest groups are also presented: the symbol * indicates p < 0.05 for the comparison of the groups to the control group, the symbol + indicates p < 0.5 for the comparison of the groups to each other. In panel (**b**), the trend of the combined parameter is provided, separating CaCO_3_ (left image) and HA (right image). Since agarose values were much higher than HA ones (at least a difference of one order of magnitude), they were excluded from HA representation. Finally, panel (**c**) shows the regression analysis for both CaCO_3_ (left) and HA (right) at different concentrations.

Aiming to the definition of a comprehensive index characterizing bone-mimicking phantoms without the need for reference measurements, a combination of the SampEn, peak1, and peak2 parameters was investigated according to Eq. (3). This equation results from a thorough regression analysis, also varying the exponent of the power, whose goodness-of-fit results are reported in the supplementary material Tables S1, S2, Figure S3. Figure 7 shows the trends and boxplot statistics for the regression analysis using f(s) (Eq. 3) for Ag 2% (control). The model presented an exponentially decreasing trend with increasing concentrations, further confirmed by the regression analysis. Since values obtained with CaCO_3_ samples were much higher with respect to those obtained from HA values (see Fig. 7, panel a) they were also displayed independently in two different scales (second-row Fig. 7). The regression analysis based on an exponential model was also computed separately for CaCO_3_ and HA, obtaining a R^2^ value of 0.6375 and 0.7249, respectively. The relatively low values of R^2^ obtained can be due to the large data dispersion, especially for Ag 2%. The control values (Ag 2%) were one order of magnitude higher than HA values. Hence they were not considered in the HA regression.

The post-hoc analysis from Mann–Whitney tests were also performed and showed in the supplementary material Table S2. All the possible pairs of concentrations were tested for the model f(s) at a significance level of 0.5.

## Discussion

In this study, we investigated the possible use of the raw RF signals for extracting information about bone mineral composition during the fracture healing process. Hence, mimicking bone-phantoms containing HA and CaCO_3_ particles at different concentrations were first prepared chemically and then scanned with US.

In Fig. 5 agarose hydrogels showed acoustic properties and echogenicity similar to water, as proved in^26^. Increasing the concentration of particles included in the agarose matrix, in addition to an higher attenuation within the sample due to absorption and scattering phenomena, a large part of the energy is reflected back at the first interface (see HA 50% in Fig. 5). Hence, the echogenicity within the sample at the highest concentration (e.g., HA 50%) was observed to be very similar to Ag 2% and water. Since B-mode images can provide only qualitative information about the material composition, we focused on the analysis of RF data and in particular we investigated the SampEn of the phase signal. In Fig. 6, SampEn results were reported, showing lower values at lower concentrations of particles. The inclusion of particles within the agarose matrix can change the phase of the signal and thus the irregularity of its time series (i.e., SampEn). The highest and more similar values of SampEn were associated with the two extreme concentrations of particles: 0% (Ag 2%) and 50% (HA 50%). Although this behavior may seem confusing, also the morphology shown by SEM images in Fig. 4 (left panel) demonstrated a similar (compact) structure for Ag 2% and HA 50%, especially at 700X magnification. Moreover, this result can be related to the different levels of energy crossing the samples, due to the different reflections at the top and bottom interfaces. Indeed, as the SampEn parameter is calculated from the phase of the RF signal in the time-domain, it does not take into account changes in the signal amplitude. To consider the impact of this phenomenon, we calculated the maximum amplitude of reflection at the first and second interfaces. The parameters peak1 and peak2 changed according to an expected trend (see Fig. 6): the amplitude of the first reflection increased with increasing concentration due to more significant differences in acoustic impedances, whereas the amplitude of the second reflection decreased with increasing concentration because of less ultrasound energy that crosses the sample.

Very few studies have been conducted concerning QUS in the context of bone fracture. The US data from bone were mainly processed to analyze changes in the SoS during callus development^18^. Other few studies focused on the characterization of tissue components for orthopedic applications. Gudur et al.^20^ explored the high-frequency spectral parametrization for the characterization of collagen hydrogels doped with HA particles. Their results demonstrated that the MBF corresponded to HA concentration and thus, it could be used to characterize the distribution of mineral content in the construct. Mercado et al.^21^ investigated the IBC (an estimation of the backscattered intensity) for the characterization of collagen-based hydrogels. They found that the IBC increased linearly with increasing collagen concentration, indicating that collagen can act as an acoustic scatterer in acellular constructs. However, in all the mentioned studies, a reference signal was needed to normalize the tissue signal and to remove artifacts associated with the US system. At a clinical level, such a normalization process requires the use of a reference phantom with acoustic properties that are known and similar to the investigated tissue in order to calibrate the measurement system^22^.

A substantial advantage of the method proposed in this study is that it does not need such a normalization step. To the best of our knowledge, the proposed method is the first combining RF phase entropy and amplitude information, thus weighing SampEn parameter through a sort of transmission index ($\frac{peak2}{peak1}$). The results in Fig. 7 showed an evident exponential decay from 0 to 50% of particles in the agarose matrix, also confirmed by the regression results. The exponent equal to 4 in the Eq. (3) was chosen according to the results of the regression analysis presented in the supplementary material Table S1, Figure S3. The regression analysis for the aggregated index f(s) (Eq. 3) was validated with R^2^ statistics as high as 0.7249. This result is very satisfactory considering the high inter-, and intra-sample variability, as well as the absence of reference sample statistics. The potential of this new model in discriminating the different concentrations of particles was also evaluated.

In Table S2 (see Supplementary Material), the results of the statistical comparison showed excellent statistical behavior associated with the model f(s). More in detail, the results between all the possible pairs of concentrations demonstrated the ability of the model f(s) to discriminate most of the tested concentrations, even ones that were close to each other. No statistically significant differences were found only between the groups CaCO_3_ 4%, CaCO_3_ 6% and the groups HA 20% and HA 50%. These results reflect those obtained from the microanalysis in Fig. 4 (right panel). Indeed, the content of Ca was comparable for CaCO_3_ 2% and CaCO_3_ 4% samples. Similarly, we observed a coherent behavior in the content of P and Ca for HA 20% and HA 50%.

We used SampEn to estimate the entropy of the RF instantaneous phase. We are aware that SampEn is one of the many indices quantifying entropy in a series generated by dynamical systems. We chose SampEn because it is an improved version of the so-called approximate entropy^29^. Previous studies using RF data exploited the theoretical definition of Shannon entropy. In^30^ the Shannon entropy performance was evaluated for the assessment of fatty liver diseases. The authors showed that fatty infiltration increased the uncertainty of the backscattered signal, thus leading to higher values of entropy. Tsui et al*.*^31^ worked to increase the resolution of entropy imaging by reducing the size of the window. The results showed a higher performance of small-window entropy imaging compared with the Nakagami parametric imaging for breast tumor classification. Tsui et al*.*^32^ also proposed a weighted entropy method using signal amplitude as weighted factors. They demonstrated the advantages of the weighted entropy with respect to the standard Shannon entropy (calculated using the raw RF data and not envelope data) for detecting the number density of scatterers in a scattering medium. Klimonda et al*.*^33^ developed multi-parametric classifiers using weighted entropy, shape parameters and texture parameters extracted from RF signals and assessed their ability to distinguish between malignant and benign tumors. Entropy images from envelope data showed higher performance than conventional methods in risk evaluation for metabolic syndrome in patients with nonalcoholic fatty liver disease^34^. On the other hand, Fang et al*.*^35^ applied Shannon entropy to US parametric imaging based on log-compressed backscattered signals, obtaining good results in assessing hepatic steatosis.

It is important to remark that previous works focused on the computation of entropy on either raw backscattered data or the envelope data. In this study, we investigated the phase content of the RF signal by calculating the SampEn parameter using the phase of the backscattered signals. The instantaneous phase of the RF signal reflects the continuity of the reflection waveforms, which characterizes the tissue composition. By exploiting the theory of complex systems, we used SampEn to quantify the irregularity of the instantaneous phase series and combined it with other features defined in the time domain for an effective, reference-free tissue characterization. As far as we know, only one study has been conducted aimed at exploring the complexity of phase US signal^36^. The authors analyzed the distribution of the phase differences in US data acquired from areas of the premature baby’s brain tissue. This information was used to create entropy images, which showed the possibility of identifying anatomical tissue structures even if qualitatively. Thus the aim and the methods used in the above paper were very different from the ones proposed in this study.

The proposed method, including SampEn and amplitude reflection information, demonstrated the capability to discriminate different mineral concentrations, which are typical of bone healing phases after a fracture. The healing of bone fracture is a continuous process that evolves through three main phases in which the bone callus composition changes^37^. Indeed, the bone mineral matrix evolves, leading to an increase in HA and CaCO_3_ content. An objective parameter able to discover these composition changes may help the surgeons to follow the healing process in a quantitative and non-invasive way, also giving the possibility to prevent problems related to incomplete healing. To the best of our knowledge, this is the first time that the phase entropy of the backscattered US signals is explored as a metrics for monitoring changes in bone mineral content.

In-vitro models are intrinsically limited, due to the impossibility to reproduce the complexity of full natural tissues. In this study, we focused on the main mineral components of the bone callus, but other elements are also present, such as organic matrix elements (mainly type I collagen) and blood. Hence, the in-vivo translation of this technique implies some challenges (such as the high inhomogeneity of natural tissues) which have to be faced in future ex-vivo and in-vivo experiments to assess the suitability of this approach in the clinical scenario. On the other hand, the system that we used in our study presents some features that are promising in view of a future in-vivo application. First, we used a certified clinical probe with a frequency range commonly used in diagnostic sonography. Second, the acquisition system allows to easily select the region of interest of a target tissue from B-mode images, thus acquiring RF data selectively. So, whenever a target tissue can be displayed by B-mode images, it is possible to acquire RF data in a specific region of interest of such tissue. The acquired RF data can then be investigated offline to extract the metric of interest and last to give quantitative data to the physician.Third, in our proposed method of analysis the commonly used normalization step by using a reference signal is not needed. This could simplify and speed up the in-vivo application: indeed the US system has not to be equipped with a calibration phantom specific for each clinical treatment. Moreover, this approach may be extended in the future to the assessment of other tissues, e.g., cartilage. This would enable to verify cartilage status at different levels of traumas or degenerative diseases (such as osteoarthritis) and to have a metrics to evaluate tissue healing overtime after a treatment, without recurring to the current gold standard methods, namely radiographic images^38^.

## Conclusion

In this work, in vitro experiments were conducted to investigate the performances of ultrasound phase entropy in detecting changes in bone mineral content, such as hydroxyapatite and calcium carbonate. For this purpose, we developed and validated an ad-hoc experimental setup using known materials. Then, bone-mimicking phantoms with increasing particle concentrations were prepared and successfully tested using SEM microscopy and EDX spectroscopy. The analysis of RF data indicated that the phase of backscattered signals from mineralized constructs contain essential information about the internal microstructure of the samples. We proposed a new model based on a combination of phase entropy information and amplitude information. This metric was able to discriminate most of the tested mineral concentrations, showing a clear trend with respect to the particle concentrations. The proposed method avoids the normalization step, proving advantages for the in-vivo translation. Overall, these results pave the way for the use of quantitative ultrasound in the diagnosis and monitoring of bone fracture healing. Besides, the proposed method could also be applied in the future for the quantitative assessment of other degenerated tissues (such as the cartilage during the osteoarthritis process), that can be displayed by US imaging.

## Supplementary Information

Supplementary Information

## Data Availability

The datasets generated during and/or analysed during the current study are available from the corresponding author on reasonable request.
