# Supplementary material for: A novel quantitative and reference-free ultrasound analysis to discriminate different concentrations of bone mineral content
Source: Sci Rep. 2021 Jan 11;11:301. doi: 10.1038/s41598-020-79365-0 (PMC7801603; doi:10.1038/s41598-020-79365-0)
Supplement: Supplementary file 1 — Supplementary Information [file 41598_2020_79365_MOESM1_ESM.pdf]

SUPPLEMENTARY MATERIAL

A novel quantitative and reference-free ultrasound analysis to discriminate different concentrations of bone mineral content

A. Sorriento[1,2], A.Poliziani[1,2#], A. Cafarelli[1,2#], G.Valenza,[3,4],L. Ricotti[1,2]*

[1]The BioRobotics Institute, Scuola Superiore Sant'Anna, 56127 Pisa, Italy

[2]Department of Excellence in Robotics & AI, Scuola Superiore Sant'Anna, 56127 Pisa, Italy

[3]Bioengineerring and Robotics Research Centre E Piaggio, University of Pisa,56122 Pisa, Italy

[4]Department of Information Engineering, University of Pisa, 56123, Pisa, Italy

*Correspondence to [angela.sorriento@santannapisa.it]

[#]These authors share equal contribution

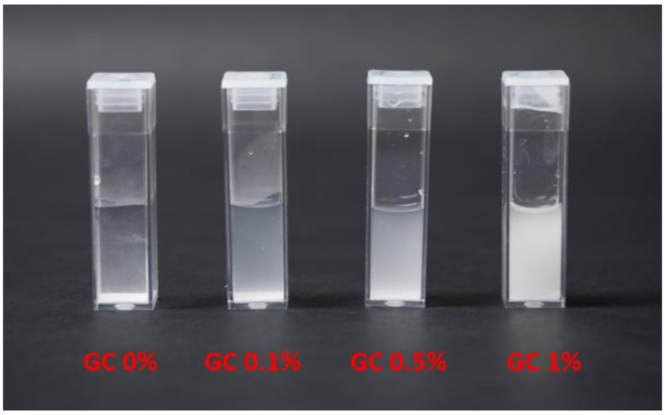

Figure S1 - Pictures for particle dispersion at different coating concentrations. *The picture was taken 30 min after sonication, showing the ability of different GC concentrations (0%, 0.1%, 0.5% and 1%) to promote and maintain overtime particle dispersion.* $CaCO_3$ *particle dispersion considerably improved when higher concentrations of GC were employed.*

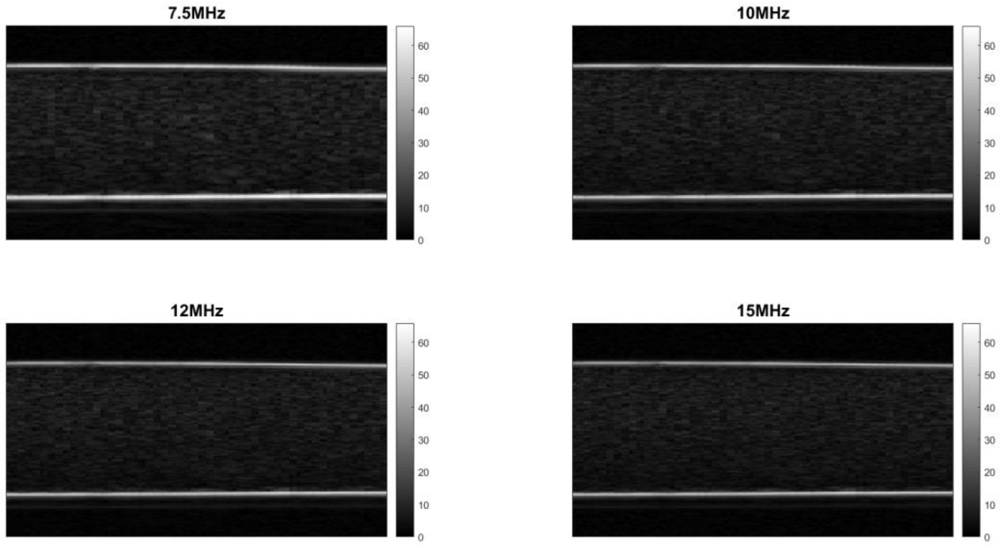

Figure S2 - B-mode images at different transmission frequencies for one representative sample of one representative experimental group. *The B-mode images of one representative sample of HA 10% are reported for each transmission frequency provided by the acquisition system (7.5MHz, 10MHz, 12MHz, 15MHz). A slight change in the resolution can be observed with increasing the transmission frequency: a more focused image is obtained using data acquired at 15MHz. It can also be noted as the brightness values are higher at lower transmission frequencies, because of less signal attenuation. As a higher frequency is associated with a higher resolution, we decided to choose RF data acquired with the transmission frequency of 15MHz for all the analyses.*

$(sampEn)^n \times \frac{peak2}{peak1}$						
	n=1	n=2	n=3	n=4	n=5	n=6
R^2 (Ag-$CaCO_3$)	0.3394	0.4850	0.5675	0.6375	0.6795	0.7075
R^2 (HA)	0.9043	0.8399	0.7965	0.7249	0.6130	0.4476
**mean R^2**	0.5815	0.6371	0.6656	**0.6750**	0.6510	0.5961

Table S1 - R-square results when the exponent value of the model was varied from 1 to 6. *The regression analyses were performed separately for CaCO3 and HA. Since agarose values were much higher than HA values (at least one order of magnitude), they were not included in the HA analysis. A mean R-square value was calculated between the two components, considering the presence of 4 independent variables for CaCO3 and 3 independent variables for HA. The maximum mean R-square value was found for the exponent 4.*

Note: the weighted average value (mean R^2) among the two regression analysis was calculated following the formula: mean R2 $= \frac{\text{R2 (Ag}-\text{CaCO3)}*4+\text{R2 (HA)}*3}{7}$

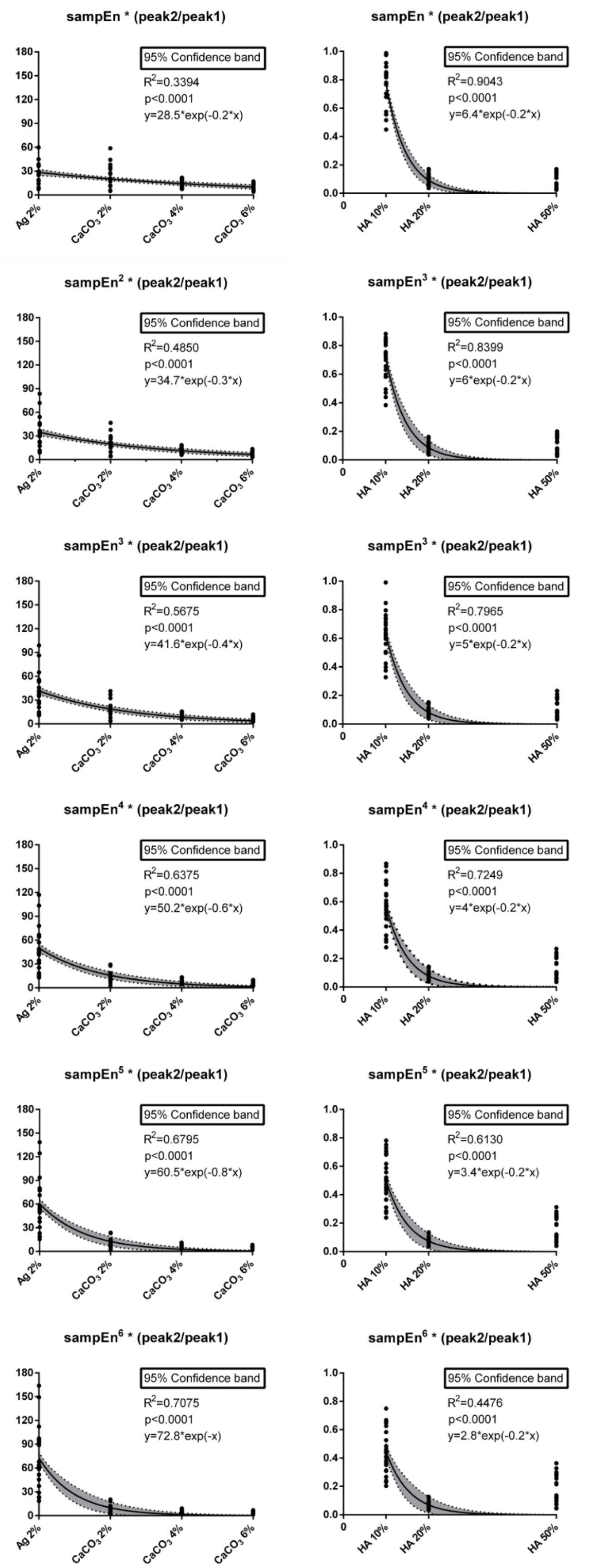

Figure S3-Regression analysis results when the exponent of f(s) was varied from 1 to 6. *The regression analyses were performed independently for CaCO3 and HA. Since agarose values were much higher than HA values (at least one order of magnitude), they were not included in the HA analysis. 25 values for each group were considered. Outliers were excluded from the analyses. The p*

value was the result of the extra sum-of-squares F test used to decide whenever the exponential model improved the model's fit with respect to a constant model (regression coefficient equal to 0). The alfa level for such a test was fixed at 0.05.

Statistical significance						
$y=sampEn^n * (peak2/peak1)$						
**Groups**	**n=1**	**n=2**	**n=3**	**n=4**	**n=5**	**n=6**
**Ag 2%-CaCO3 2%**	ns	*	*	*	*	*
**Ag 2%-CaCO3 4%**	*	*	*	*	*	*
**Ag 2%-CaCO3 6%**	*	*	*	*	*	*
**Ag 2%-HA 10%**	*	*	*	*	*	*
**Ag 2%-HA 20%**	*	*	*	*	*	*
**Ag 2%-HA 50%**	*	*	*	*	*	*
**CaCO3 2% -CaCO3 4%**	+	+	+	+	+	+
**CaCO3 2% -CaCO3 6%**	+	+	+	+	+	+
**CaCO3 2% -HA 10%**	+	+	+	+	+	+
**CaCO3 2% -HA 20%**	+	+	+	+	+	+
**CaCO3 2% -HA 50%**	+	+	+	+	+	+
**CaCO3 4%- CaCO3 6%**	ns	ns	ns	ns	ns	ns
**CaCO3 4%-HA 10%**	+	+	+	+	+	+
**CaCO3 4%-HA 20%**	+	+	+	+	+	+
**CaCO3 4%-HA 50%**	+	+	+	+	+	+
**CaCO3 6%-HA 10%**	+	+	+	+	+	+
**CaCO3 6%-HA 20%**	+	+	+	+	+	+
**CaCO3 6%-HA 50%**	+	+	+	+	+	+
**HA 10%-HA 20%**	+	+	+	+	+	+
**HA 10%-HA 50%**	+	+	+	+	+	+
**HA 20%-HA 50%**	ns	ns	ns	ns	+	+

Table S2 - Statistical results when the exponent value of the model was varied from 1 to 6. *A two-sided Wilcoxon rank-sum test with a significance level of 0.05 was used. All the possible pairs of concentration were compared. ns=non-significant, *=p<0.05 for the comparison of the groups to the control group, +=p<0.5 for the comparison of the groups to each other.*
